# Algal Foams Applied in Fixed-Bed Process for Lead(II) Removal Using Recirculation or One-Pass Modes

**DOI:** 10.3390/md15100315

**Published:** 2017-10-17

**Authors:** Shengye Wang, Thierry Vincent, Catherine Faur, Eric Guibal

**Affiliations:** 1 IMT Mines Alès, Materials Research Center, 6 Avenue de Clavières, F-30319 Alès CEDEX, France; Thierry.Vincent@mines-ales.fr; 2 European Institute of Membranes, IEM (UMR-5635, University of Montpellier, ENSCM, CNRS), Place Eugène Bataillon, 34095 Montpellier CEDEX 5, France; Catherine.Faur@umontpellier.fr

**Keywords:** lead, fixed-bed column, alga, foam, sorption, desorption

## Abstract

The incorporation of brown algae into biopolymer beads or foams for metal sorption has been previously reported. However, the direct use of these biomasses for preparing foams is a new approach. In this study, two kinds of porous foams were prepared by ionotropic gelation using algal biomass (AB, *Laminaria digitata*) or alginate (as the reference) and applied for Pb(II) sorption. These foams (manufactured as macroporous discs) were packed in filtration holders (simulating fixed-bed column) and the system was operated in either a recirculation or a one-pass mode. Sorption isotherms, uptake kinetics and sorbent reuse were studied in the recirculation mode (analogous to batch system). In the one-pass mode (continuous fixed-bed system), the influence of parameters such as flow rate, feed metal concentration and bed height were investigated on both sorption and desorption. In addition, the effect of Cu(II) on Pb(II) recovery from binary solutions was also studied in terms of both sorption and desorption. Sorption isotherms are well fitted by the Langmuir equation while the pseudo-second order rate equation described well both sorption and desorption kinetic profiles. The study of material regeneration confirms that the reuse of the foams was feasible with a small mass loss, even after 9 cycles. In the one-pass mode, for alginate foams, a slower flow rate led to a smaller saturation volume, while the effect of flow rate was less marked for AB foams. Competitive study suggests that the foams have a preference for Pb(II) over Cu(II) but cannot selectively remove Pb(II) from the binary solution.

## 1. Introduction

Release of Pb(II) to the environment has caused widespread public concern due to its high toxicity and un-biodegradability [1]. A variety of removal technologies such as precipitation, membrane separation, solvent extraction have been widely investigated, but these methods are plagued since they are either economically impractical or producing huge amounts of secondary waste [2]. Biosorption process has been recently considered as an alternative technique due to its low cost, ease of operation and high efficiency. Many different types of materials have been considered for the development of biosorbents, including agriculture wastes, seaweed biomass, biochar, biopolymers, etc. Most of them are grinded to millimeter-scale or micrometer-scale powders to achieve larger surface areas and faster removal, but the difficulty in recovering such materials from the treated water is very likely to cause environmental concerns and difficulties in processing, which, in turn may affect their reusability [3]. Therefore, magnetically modified sorbents that present good separation performance have been developed more recently [4]. However, these sorbents still have disadvantages: they are usually not stable in acid solutions. For example, iron ions may be significantly released from magnetic biochar when the pH of the solution is below 3 [5]; this restricts the use of strong acids in metal desorption process. Moreover, for practical application, continuous sorption studies in packed bed columns are more useful [6], but these materials are generally difficult to be used in such systems due to head loss and clogging effects.

These problems can be addressed by shaping the sorbents under the form of porous beads or foams with a macroporous structure; many examples have been reported using biopolymers such as chitosan, or alginate. However, a wide application of such sorbents will result in an increase of production of these biopolymers, which will undoubtedly bring new environmental concern. Indeed, the extraction/purification processes involved in their production require using more reagents and induce the production of poorly valorizable residues; this may cause negative environmental impact and also increase the cost of the sorbents. For these reasons, directly using the algal biomass, instead of the extracted alginate, sounds to be a promising green and competitive process for developing alternative biosorbents. While most of the studies encapsulated the powders into pure biopolymers [7], our previous study proposed a one-pot method for the preparation of algal beads without adding any pure polymers [8,9]. This environmental-friendly method consists of the mild-alkaline extraction of alginate-based compound from the algal biomass and the further distribution of the as-prepared suspension into an ionotropic gelation solution, avoiding using chemical agents for purification process. This “one-pot synthesis” concept can also be used for preparing foam discs. These foam-discs can be easily packed in filtration equipment and applied in fixed-bed system. In addition, when packed in a filtration holder, these materials neither are subjected to intense shaking nor to high-speed agitation; this makes the sorption systems more stable.

In this study, the foams prepared as above were packed in filtration holders for the removal of Pb(II) using recirculation or one-pass modes. In the former mode, sorption isotherms, uptake kinetics under different flow rates and sorbent regeneration were investigated, while in the latter mode, the effect of velocity, feed metal concentration and bed height (number of discs arranged in-series) was studied. In addition, the sorption and desorption of Pb(II) in Pb(II)-Cu(II) system were carried out in one-pass mode to assess the potential of removing Pb(II) from a multi-metal solution and to check the concentration effect.

## 2. Results

### 2.1. Characterization

**Figure 1:**
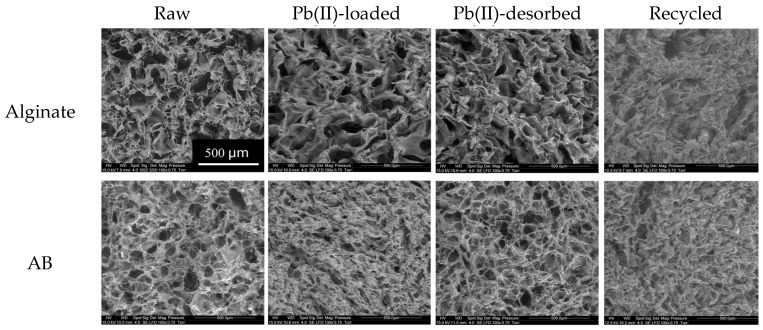
Scanning electron microscopy (SEM) micrographs of raw, Pb(II)-loaded, Pb(II)-desorbed and recycled alginate and AB foams.

The porosity, bulk density, pH_PZC_ (point of zero charge) and thickness of the foams have been determined and the results can be found in Table S1 (see Supplementary Materials). The pH_PZC_ was determined by the pH-drift method. This corresponds to the charge neutralization at the surface of the sorbent (see Supplementary Materials). SEM micrographs (Figure 1) show that both alginate and AB (Algal biomass) foams exhibit high toughness and a large internal porosity all over the whole surface, guaranteeing their good percolating property. In addition, the structure of Pb(II)-loaded foams appears to be more compact and some small holes become invisible. This trend is less marked after Pb(II) desorption. This can be explained by the fact that ionic radius of Pb(II) (0.98 Å) is greater than that of Ca(II) (0.78 Å), so generally, the coordination sphere of Pb(II) embraces a larger number of carboxyl groups than that of Ca(II) [10]. Therefore, the polymer chains are more twisted in the Pb(II)-bound matrix. After 9 sorption/desorption cycles, the surface of the foams becomes less rugged. This is probably due to the little mass loss of material during the succession of sorption and desorption cycles (see below Section 2.2.3).

Figure S1 (see Supplementary Materials) represents the EDX analysis of the original, Pb(II)-loaded and recycled (9 sorption/desorption cycles) foams. The raw material is characterized by the presence of C, O and Ca elements, which are considered as common elements existing in calcium alginate beads (as constituent of the organic structure of the biopolymer for C and O elements, and representative of the ionotropic gelation agent for Ca element). After Pb(II) sorption, the most significant change is associated to the replacement of Ca peaks on the EDX analysis with the signals of Pb element; the sorption mechanism of Pb(II) involves ion exchange between Pb(II) and Ca(II) ions. Contrarily, after desorption process, Ca element re-appears in the foams (acidic CaCl_2_ solution was used as the eluent) while the intensity of Pb element peaks largely reduces (the element is still detected since the efficiency of desorption is lower than 100%, see below). Similar trends are observed in the case of recycled foams.

The analysis of these materials (raw, Pb(II)-loaded, Pb(II)-desorbed and recycled foams) was also performed by FTIR spectroscopy (Figure S2, see Supplementary Materials, wavenumber range: 1800–400 cm^−1^). The assignments of the main bands in spectra are reported in Table S2 (see Supplementary Materials). The Pb(II)-free spectra of the alginate and AB foams present very similar profiles. The broad absorption peaks observed between 3000 and 3500 cm^−1^ and around 2928 cm^−1^ represent –OH and C–H stretching vibration, respectively. The peaks at 1650–1580, 1429, 1025 and 810 cm^−1^ correspond to COO– asymmetric stretching [11], COO– symmetric stretching [12], C–O–C anti-symmetric stretching [13] and CH_3_ rocking vibration [14], respectively. It is noteworthy that although the alga contains a certain amount of fucoxanthin and protein, the peaks corresponding to sulfonate (on fucoxanthin) and amine groups (on protein) vibration are not detected on AB foams, which can be explained by the weakness of their signals. Indeed, the fucoxanthin and protein contents on *L. digitata*, are reported to be as low as 0.16–0.47% and 1.5–5.1% (depending on the sites where they were collected), respectively [15].

After Pb(II) binding, the main changes are associated to a slight shift of the peaks from 3240 cm^−1^, 1592 cm^−1^ and 1415 cm^−1^ (assigned to –OH stretching, COO– asymmetric and asymmetric stretching, respectively) to 3209 cm^−1^, 1568 cm^−1^ and 1404 cm^−1^ for alginate; similar trend is found for AB foams, indicating that hydroxyl and carboxyl groups in the foams are involved in Pb(II) sorption, as the main functional groups.

For the foams after Pb(II) desorption, many changes suggest that the sorbents are chemically modified by the contact with hydrochloric acid. A new peak is appearing at 1728 cm^−1^ for alginate and 1723 cm^−1^ for AB foams, assigned to the carbonyl stretching mode of carboxylic acid groups, while the COO– peak either shifts or completely disappears; this indicates that a part of carboxyl groups on the foams are converted to their anhydride form [16]. Other changes consist of the appearance of a band at 1260 cm^−1^ (which corresponds to CH_3_ symmetric bending) and a shift of peak in CH_3_ rocking region. Despite a very slight shift, the peaks in the recycled materials (recycled 9 times) are almost the same with Pb(II)-desorbed material (recycled once), showing the stability of both the pristine reactive groups and the new groups that appear after the first desorption process. As discussed above, most of Pb(II) sorption occurs via coordination to carboxyl groups; the succession of sorption/desorption processes, to some degree, changes the chemical structure of the foams. The experiments of sorbent reuse will show if this change affects the sorption performance (see below, Section 2.2.3).

### 2.2. Recirculation Mode

#### 2.2.1. Sorption Isotherms

The study of sorption isotherm is of fundamental importance in (a) understanding the solute distribution between the liquid and solid phases, (b) determining the maximum sorption capacity of the sorbents and (c) evaluating their affinity for target metal ions. In this study, the recirculation mode is applied to investigate the sorption isotherms and linear and non-linear Langmuir equations are used for fitting the experimental data. Figure S3 (see Supplementary Materials) shows that the experimental profiles are fitted well by linear Langmuir equation with a coefficient of determination (R^2^, shown in Table S3, see Supplementary Materials) of 0.999 (both for alginate and AB foams). The maximum sorption capacities reach 2.26 mmol Pb g^−1^ for alginate foam and 1.22 mmol Pb g^−1^ for AB foam; these values are close to those obtained from experimental data (2.24 and 1.22 mmol Pb g^−1^, respectively). Affinity constant (*b*) represents the stability of the bonds formed during the sorption reactions [17]. The affinity coefficients calculated for both foams are very close (193 and 205 L mmol^−1^ for alginate and AB foams, respectively). The best sorbent cannot be selected on the basis of metal affinity, contrarily to maximum sorption capacities (more favorable to Alginate foams).

The carboxyl groups on alginate are the main functional groups involved in Pb(II) binding (as discussed in Section 2.1). However, although the alginate content in algal biomass (*L. digitata*) is only around 31% (the amount of alginate is three times that of AB foams), the Pb(II) sorption capacity of alginate foams is less than twice than that of AB foams. The reason has been fully discussed in the previous study using the same materials conditioned under the form of spherical beads [18]. Briefly, a better dispersion of alginate in AB due to the presence of other constituents (such as cellulose fibers) and less compact structure of AB foams could contribute to enhancing the accessibility and reactivity of carboxylic groups on the chain and thus improving the sorption property. Moreover, for alginate sorbent, the functional groups are carboxyl groups and the sorption mechanism is limited to ion-exchange between Pb(II) and the protons on carboxylic groups or calcium ions bound to carboxyl groups, while in the case of AB, beside the carboxyl groups, the presence of a small amount of sulfonic acid groups on fucoidan (sulfated polysaccharide, RO-SO_3_^−^) and amine groups on proteins could also play a role in binding Pb(II).

#### 2.2.2. Sorption/Desorption Kinetics

**Figure 2:**
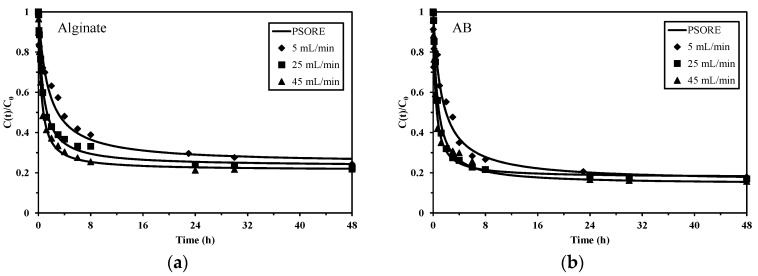
Sorption kinetics using (**a**) alginate and (**b**) AB foams at different flow rates—Modeling of uptake profiles with the pseudo-second order rate equation (PSORE) (pH 4.0; C_0_: 0.5 mmol Pb L^−1^; V: 500 mL; mass (dry weight): 100 mg for alginate and 200 mg for AB foams).

The sorption kinetics can be controlled by reaction rate, mass transfer but also pore filling for those porous sorbents. For the recirculation mode in fixed-bed column system, the flow rate also plays an important practical role on uptake kinetics, through its effect on contact time. Especially for those gel-type sorbents, a high flow rate will result in a short contact time: which consequently may result in the inability to access to all the reactive sites in the core of the composites (due to mass transfer resistance) [19]. However, providing a sufficient flow rate for the reactor allows decreasing the impact of resistance to bulk diffusion and to film diffusion. Three different flow rates (5 mL min^−1^, 25 mL min^−1^ and 45 mL min^−1^, which are corresponding to superficial velocities of 0.61 m h^−1^, 3.06 m h^−1^ and 5.51 m h^−1^, respectively), were compared for the kinetic profiles (evolution of the residual Pb(II) concentration in the reactor). The experiments were carried out in the recirculation mode for a total of 48 h, to ensure that the sorption equilibrium is completed. Figure 2 shows that for alginate, the increase of the flow rate leads to a steeper decrease of residual metal concentration, while for AB this trend is less marked, especially when the flow rate is higher than 25 mL min^−1^. These results can be explained by the fact that although a slower flow rate provides more contact time in the bed, an increase of the flow rate on the other hand, helps in eliminating air bubbles (which may limit the contact of the solution with reactive groups at the internal surface) in the foams and in forcing the hydrodynamic transfer of the solution in their whole mass [20]. Different relative fractions of G and M units and the presence of cellulose fibers in algal biomass may contribute to facilitate the mass transfer, requiring a slower flow rate for AB than the pure polysaccharide foams for achieving fast diffusion. The results also confirm that a flow rate of 25 mL min^−1^ is sufficient for AB foams to ensure that all the sorption sites are made readily available for Pb(II) uptake.

The pseudo-second order rate equation (PSORE) fitted the experimental profiles better (Figure 2) than pseudo-first order rate equation (PFORE). This conclusion can also be confirmed by comparing the coefficient of determination, R^2^, obtained for the two models (taking flow rate = 45 mL min^−1^ as an example) as shown in Table 1. Moreover, the predicted equilibrium sorption capacity (q_eq,calc._) from PSORE for both alginate and AB foams are close to those obtained from experiment (q_eq,exp._). While Seki et al. [21] reported a good fit of kinetics of Pb(II) sorption onto biopolymer composites (association of alginic acid and humic acid) by the PFORE, most of the researchers [22,23] concluded that the sorption of Pb(II) onto alginate-based sorbents follows the PSORE. The apparent rate coefficients (k_1_) range for PFORE between 28.3 × 10^−3^ min^−1^ and 34.1 × 10^−3^ min^−1^, for alginate foam and AB foam respectively. For PSORE, the rate coefficients (k_2_) vary between 20.1 × 10^−3^ g mmol^−1^ min^−1^ and 44.2 × 10^−3^ g mmol^−1^ min^−1^, respectively. For both PFORE and PSORE, the rate coefficients are higher for AB foam than for alginate foam. It is difficult to compare the k_2_ values with those in the literature since it can be affected by many experimental conditions (initial concentration, solution pH, sorption systems, etc.) [24]. To conclude, the sorption kinetics using different flow rates obey PSORE, suggesting that the sorption of Pb(II) onto alginate and AB foams mainly occurs through chemisorption; ion exchange takes place essentially between Pb(II) and Ca(II) on the carboxyl groups [25].

**Figure 3:**
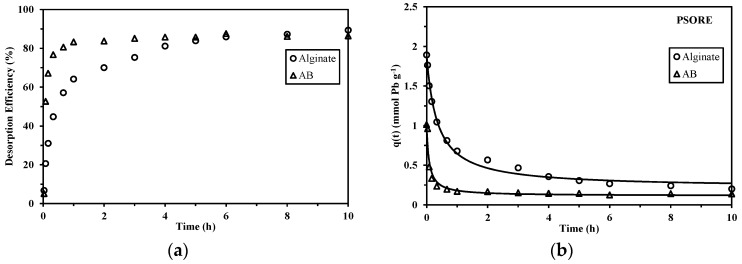
Desorption kinetics of alginate and AB foams at 25 mL min^−1^: (**a**) desorption efficiency, and (**b**) Modeling of kinetic profiles with the PSORE (V: 500 mL; flow rate: 25 mL min^−1^; desorption agent: 2 M HCl/0.05 M CaCl_2_; Pb(II)-loaded sorbent obtained from sorption kinetics at 25 mL min^−1^).

The desorption kinetics were conducted at a flow rate of 25 mL min^−1^ using acidic calcium chloride as the desorption agent. Figure 3 shows that, in general, AB foams present a faster desorption than alginate foams: for AB sorbent, equilibrium has been obtained within the first hour, while for alginate sorbent, it requires at least 6 h. This again can be explained by the presence of cellulose fibers in AB foams, which facilitates mass transfer. At equilibrium, the desorption efficiency is close to 89.4% and 87.6% for alginate and AB foams, respectively. The uncomplete desorption of Pb(II) could be due to the high affinity of alginate for Pb(II) [26]. Vijayalakshmi et al. [27] observed a Pb(II) desorption efficiency of 75.1% from nanochitosan/sodium alginate/microcrystalline cellulose beads with a contact time of 30 min. Similarly, Soltani et al. [28] reported that during the experiments of sorbent (silica nanopowders/alginate composite) reuse, the desorption efficiency was 85.5% in the first cycle and decreased to 67.3% in the second cycle. Desorption kinetics are well described using PSORE with the coefficient of determination, R^2^, of 0.992 and 0.944 (Table S4, see Supplementary Materials) for alginate and AB foams, respectively. The kinetic constant k_2d_, again demonstrates the quicker desorption of Pb(II) from AB than alginate foams: the value of k_2d_ for AB (i.e., 2.29 × 10^−1^ g mmol^−1^ min^−1^) is one order of magnitude greater than that of alginate (i.e., 2.86 × 10^−2^ g mmol^−1^ min^−1^).

#### 2.2.3. Sorption/Desorption Cycles

The reusability of the sorbents is very crucial to the economic and environmental aspects. To regenerate the sorbents after metal loading, desorption process was conducted right after the sorption step. To eliminate the residual acid in the bed after sorption process, a washing step is necessary. Yan et al. [29] rinsed the sorbents (alginate/PEI) after desorption process with deionized water until the pH was neutral; with this procedure a large amount of rinsing water would be necessary. In this study, the washing step was conducted by simply passing 50 mL of pure water through the system at a very low flow rate (0.5 mL min^−1^).

**Figure 4:**
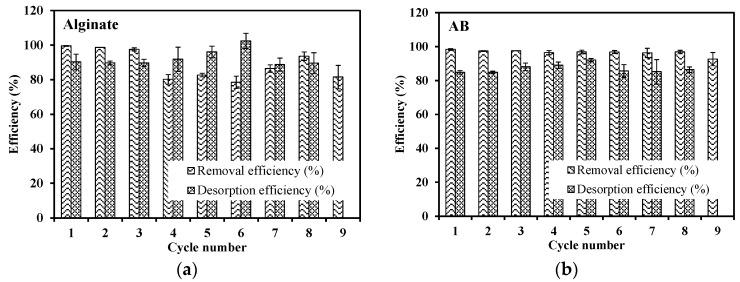
Pb(II) sorption/desorption cycles—(**a**) alginate and (**b**) AB foams recycling (Sorption step: pH 4; C_0_: 0.24 mmol Pb L^−1^ for alginate and 0.12 mmol Pb L^−1^ for AB foams; V: 500 mL; mass: 100 mg; flow rate: 25 mL min^−1^; contact time: 48 h—Washing step: 50 mL of demineralized water pumped through the foams—Desorption step: 2 M HCl/0.05 M CaCl_2_; V: 50 mL; time: 6 h; flow rate: 25 mL min^−1^).

The changes in the sorption/desorption efficiency during the 9 cycles at the 10:1 ratio of volumes used for sorption and desorption processes (i.e., V_sorp._/V_des._: 10 for increasing the concentration the eluate, compared with that in the initial solutions) are shown in Figure 4. Although the desorption efficiency for both alginate and AB foams is lower than 90%, the sorption performance maintains higher than 97% for the first 3 cycles for alginate and 8 cycles for AB foams. Indeed, these sorbents are not totally saturated under selected experimental conditions during the few first cycles: despite the uncomplete desorption of Pb(II), the active sites (regenerated and unreacted) are in excess compared to the amount of Pb(II) recirculated, meaning that free active sites are sufficient for metal binding. However, as the recycle continues, the sorption sites are progressively occupied and consequently the removal efficiency decreases. Moreover, for pure alginate foams, the acidic desorption agent may result in mass loss by solubilization and/or degradation [30]. For AB foams, cellulose-like fibers help in maintaining the structure and thus those foams present more stable sorption efficiency than alginate. Indeed, this can be demonstrated by comparing the mass loss after 9 cycles, which is 7.9 ± 1.9% for alginate, higher than 6.3 ± 0.4% for AB foams. Fagundes-Klen et al. [31] observed a weight loss of more than 20% after only 2 cycles using a modified algal biomass; this means that the present algal foams, immobilized in filter holders, are very stable. It is noteworthy that desorption efficiency increases from 4th cycle to 6th cycle for alginate and from 3rd to 5th for AB foams. This increase may be explained by the desorption of Pb(II) from previous cycles that are not completely removed at previous steps. In addition, the concentration factor (CF) is calculated as the Pb(II) concentration in the eluate (C_V_) divided by its value in the feed (C_0_). In other words, CF evaluates the performance of concentrating the metal from influent into eluate. During the sorption/desorption cycles, CF is around 7.4–9.0 and 8.2–9.0 for alginate and AB foams, respectively.

The main sorption/desorption mechanism involving Pb(II)/Ca(II) ion exchange has been confirmed by SEM-EDX results (see Section 3.1). Adding a small amount of CaCl_2_ into the desorption agent not only helps in maintaining the stability of the foams, but may also contribute to desorbing Pb(II) into the eluate. Abdolali et al. [32] also used 1 M CaCl_2_ to repair the damages caused by the desorbing agent (HCl) and thus increase the stability of modified biosorbent. Although FTIR analysis confirms that (a) the chemical structure of the foams are modified after desorption process and (b) a certain amount of carboxyl groups are converted to anhydride (see Section 3.1). The comparison of sorption and desorption performances, at the different cycles, show that the Pb(II) binding performance is not significantly changed before the first 3 cycles for alginate and 8 cycles for AB foams. This is probably due to the re-conversion of the anhydride to carboxylic acid when applying the foams in solution (contact with water). Indeed, the anhydride can be easily converted to carboxylic acid even exposed to an atmosphere containing moisture [33]. To conclude, the experiment of sorption/desorption cycles confirms the feasibility of reusing the alginate and AB foams for Pb(II) removal.

### 2.3. One-Pass Mode

To further expand the potentiality and flexibility of this type of foams applied in fixed-bed like systems, the immobilized foams were not only fed in a recirculation mode but also in a one-pass mode at a slow flow rate in order to ensure a sufficient time for the solution to be in contact with the foams. Breakthrough curves have been obtained by plotting C_v_/C_0_ vs. volume. Different parameters such as metal concentration, flow rate and bed depth (number of foams associated in series) were investigated. At last, these foams were applied in bi-metal solutions containing equimolar concentrations of Pb(II) and Cu(II).

#### 2.3.1. Effect of Velocity on Sorption/Desorption

**Figure 5:**
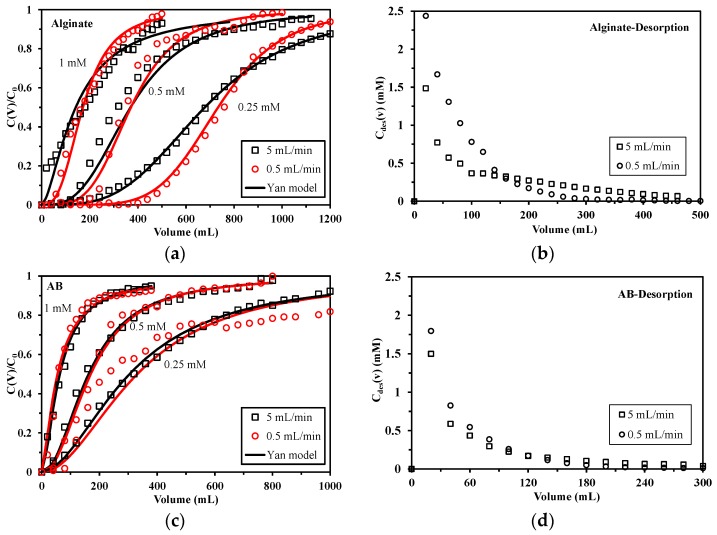
Effect of flow rate on the breakthrough curve of Pb(II) sorption onto alginate (**a**) and AB foams (**c**) (pH: 4; sorbent mass: 100 mg (i.e., 1 foam, depth = 0.32 cm for alginate and 0.26 cm for AB)), and Pb(II) desorption curves of Pb-loaded alginate (**b**) and AB foams (**d**) (Pb-loaded foams were obtained from prior sorption process).

The experiments were conducted using three different feed concentrations (C_0_: 0.25, 0.5 and 1 mmol Pb L^−1^) at two different flow rates (0.5 and 5 mL min^−1^). All the breakthrough curves of Pb(II) onto both sorbents follow the typical S-shape curve for column operation as the ratio of the effluent Pb(II) concentration (C_v_) to the input concentration (C_0_) versus volume (shown in Figure 5a,c).

It can be observed that the breakthrough curves of alginate foams are significantly affected by the flow rate: increasing the velocity reduces the steepness of the breakthrough curves. However, the effect of the flow rate is less marked in the case of AB foams, probably because of the porosity characteristic of the foams.

Table 2 indicates that for alginate foams, regardless of the feed concentration, increasing the flow rate leads to an earlier breakthrough, and faster saturation time (corresponding to smaller breakthrough volume and greater saturation volume). In other words, a higher velocity does not reduce the equilibrium sorption capacity of alginate foams, but increases the volume of effluent treated before the sorbent is saturated, resulting also in a lower removal percentage. Taking C_0_ = 0.5 mmol Pb L^−1^ as an example, the sorption capacity calculated for alginate foams is 2.10 mmol g^−1^ at 5 mL min^−1^ and 2.11 mmol g^−1^ at 0.5 mL min^−1^. This means that the Pb(II) uptake amount is directly controlled by the amount of sorbent in the bed. An opposite observation was found by Abdolai et al. [32], who reported that a higher sorption capacity of modified biosorbents for Cd(II), Cu(II), Pb(II) and Zn(II) could be achieved when reducing the flow rate. They explained that a decrease of flow rate leads to a longer residence time, and thus the intra-particle diffusion becomes more effective in controlling mass transfer. Muhamad et al. [34] reported a trend in agreement with the results observed in the present study: the flow rate poorly affected sorption capacity at equilibrium. The residence time, under selected experimental conditions is sufficient for minimizing the impact of column depth: the saturation capacity (i.e., the thermodynamic properties of the sorbent) is predominant over the diffusion properties of the sorbent in the control of overall sorption performance. The highly porous structure of alginate foams (see Section 3.1) reduces the impact of the resistance to diffusion and allows the sorbent gradually achieving saturation with continuous feed of solution. Moreover, the effect of flow rate is much less marked in the case of AB foams: at both flow rates, the breakthrough volumes remain around 40–80 mL, 40 mL and <20 mL with Pb(II) input concentration of 0.25, 0.5 and 1 mmol Pb L^−1^, respectively, and saturation volumes around 1200–1400 mL, 720 mL and 380–440 mL, respectively. This, again, can be explained by the presence of cellulose fibers in AB foams, which enhances the mass transfer properties and thus reduces the influence of flow velocity.

In addition, the Thomas [35] and Yan [36] models (described in Supplementary Materials) have been used for simulating the curves. Figure 5a,c shows that all the breakthrough curves can be fitted by Yan model regardless of feed metal concentration; the modeling curves of Thomas are not shown in the figure due to its relatively poor fitting performance. However, parameters obtained from C_0_ = 0.5 mmol Pb L^−1^ listed in Table S5 (see Supplementary Materials) suggest that although the correlation coefficients (R^2^) from the Yan model are ranging from 0.95 to 0.98, the predicted sorption capacities (q_Y_) of Pb(II) are slightly lower than the values obtained from experiments (q_e,exp._). Yan model helps to overcome some the drawbacks like serious deficiency in predicting the effluent concentration at the beginning of the breakthrough curve and shows good fitting performance, but it overestimates the values of sorption capacities in certain cases; similar conclusions were previously reported [37,38]. It should be noted that some of the experiments were stopped before the foams were fully saturated (when C_V_/C_0_ = 1) and this, to some degree, may explain the discrepancies between q_e,exp._ and q_Y_.

Desorption experiments were conducted right after the sorption process. Results in Figure 5b,d again confirm the requirement of a slow flow rate for alginate foams, particularly for the first 160 mL: while a volume of 160 mL desorbs 47% Pb(II) from alginate foams at the velocity of 5 mL min^−1^, 81% of loaded Pb(II) can be desorbed at 0.5 mL min^−1^ with the same volume of eluate. This means a smaller amount of eluate is needed for desorption step at a lower flow rate. For AB foams, the metal concentration differences in the eluate between these two velocities are much smaller, revealing a flow rate of 5 mL min^−1^ is sufficient for Pb (II) desorption step of AB foams. Reducing the volume of eluate benefits to the concentration effect of the sorption/desorption run.

#### 2.3.2. Effect of Column Depth on Sorption/Desorption

**Figure 6:**
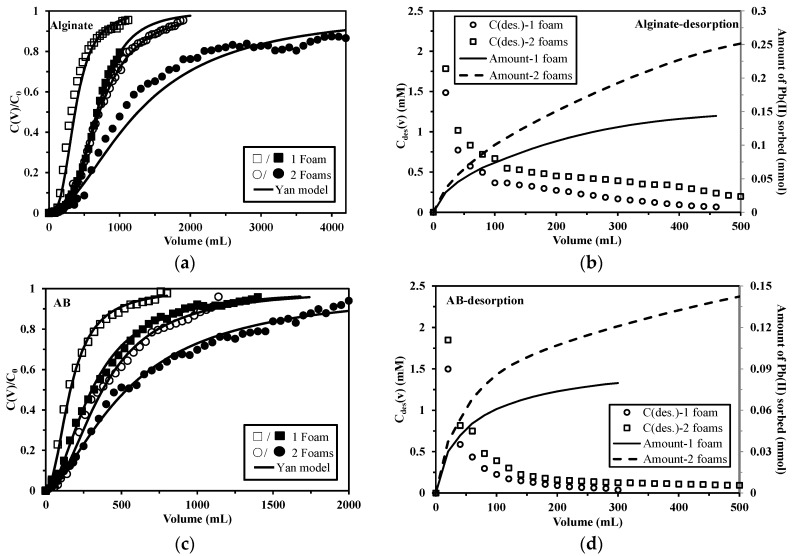
Effect of bed depth (number of foams) on breakthrough curves of Pb(II) sorption onto alginate (**a**) and AB foams (**c**). (pH: 4; influent metal concentration: 0.5 mmol Pb L^−1^ (open) and 0.25 mmol Pb L^−1^ (closed); flow rate: 5 mL min^−1^; mass = 100 mg (i.e., 1 foam, depth = 0.32 cm for alginate and 0.26 cm for AB) and 200 mg (i.e., 2 foams, depth = 0.64 cm for alginate and 0.52 cm for AB)) and Pb(II) desorption curves of alginate (**b**) and AB foams (**d**) (flow rate: 5 mL min^−1^; Pb-loaded foams were obtained from previous sorption process with C_0_ = 0.5 mmol Pb L^−1^).

Figure 6a,c shows the breakthrough curves for Pb(II) sorption onto alginate and AB foams at two different feed concentrations with different number of foams (disposed in series, representing different bed depths). The shape of the breakthrough curves changes significantly with the bed depth. The breakthrough volumes increase when the bed depth is doubled: breakthrough occurs at 120 mL and 240 mL for alginate at C_0_ = 0.25 and 0.5 mmol Pb L^−1^, respectively, when applying a single foam, while these values increase to 260 mL and 360 mL, respectively, when two foams are used. Similar trend is observed for AB foams. As expected, increasing the number of foam discs increases the number of reactive groups available for metal binding: the total surface area increases and the contact time of the solution in the column increases [39]. Consequently, the saturation volumes also increases with column depth, and the volume of treated solution almost doubled, consistently with the doubling of sorbent amount and density of reactive groups. Besides Yan model, the Adams–Bohart model (described in Supplementary Materials) [40] was also applied to describe the initial part (C_t_/C_0_ up to 0.5) of the breakthrough curves at different bed depths with different feed concentrations. This model is based on the assumption that the sorption process is continuous and that equilibrium is not instantaneously attained. The parameters, such as maximum adsorption capacity (N_o_) and kinetic constant (k_AB_), along with determination coefficients, R^2^ values, are presented in Table S6 (see Supplementary Materials). The values of kinetic constant significantly decrease with increasing bed depth. This suggests that the overall sorption kinetics are dominated by external mass transfer in the initial part of binding process [41]. The modeling curves in Figure S4 (see Supplementary Materials) shows that there is a good agreement between the experimental and predicted values, especially between the relative concentration ratio ranging from 0.1 to 0.5, suggesting that the Adams–Bohart model can be valid within this region. However, it fails to fit the whole breakthrough curves: large discrepancies are found between the experimental and predicted values when the relative concentration ratio is above 0.5 (not shown). Similar trend was reported by Han et al. [42]; this justifies the use of the Yan model.

Desorption curves are shown in Figure 6b,d. AB foams in general present a faster desorption than alginate foams regardless of bed depth; this result is in agreement with that obtained from recirculation mode and the reason has been explained above (see Section 2.3.1). In addition, for both alginate and AB foams, the metal concentration in eluate from 2 foams is higher than that from single foam and this is obviously because more metal ions were bound during sorption process when a higher bed height was applied. However, the different levels of saturation of the columns make difficult the strict comparison of sorption capacities; this is just possible concluding that the results are consistent with the amount of sorbent immobilized in the columns.

#### 2.3.3. Competitive Sorption in Pb(II)-Cu(II) System

**Figure 7:**
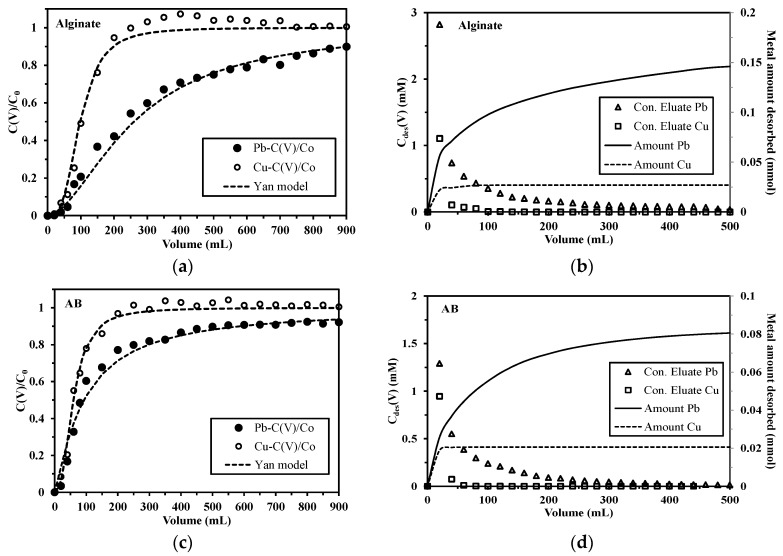
Breakthrough curves of Pb(II) and Cu(II) on alginate (**a**) and AB foams (**c**); Eluate curves of Pb(II) and Cu(II) from alginate (**b**) and AB foams (**d**) (C_0_(Pb) = C_0_(Cu): 0.5 mmol L^−1^; flow rate = 2 mL min^−1^; pH of input solution: 4; mass: 100 mg; desorption agent: 2 M HCl/0.05 M CaCl_2_).

Previous studies already published on algal and alginate beads produced with a similar basic procedure have shown the preference of these materials for Pb(II) over Cu(II) [9,18]. The objective of these tests in binary solutions with the foams consisted of verifying that in dynamic systems the sorbents conditioned as foam-shaped materials follow the same preference for Pb(II) over Cu(II). Is the conditioning changing the selectivity? The breakthrough curves shown in Figure 7a,c suggest that the sorption behaviors of Pb(II) onto both sorbents are similar to those in the mono-metal system, while those of Cu(II) are quite different: the curves after the breakthrough point are much steeper (Figure S5a, see Supplementary Materials). The C_V_/C_0_ curve for Cu(II) increases dramatically after the breakthrough of 40 mL for alginate and 20 mL for AB foams. The saturation volumes are close to 100 mL and 150 mL for alginate and AB foams, respectively, which are much smaller than those in mono-metal system (1100 mL and 1150 mL, respectively). Moreover, Cu(II) breakthrough curve shows a little overshoot at the outlet of the column, especially in the case of alginate foams. Such trend has been commonly found before, such as Cd(II) from sugarcane bagasse in competition with Cu(II) [43], Ni(II) from grape stalks waste in competition with Pb(II) [44], and Pt(IV) from chitosan derivatives in competition with Pd(II) [45]. This phenomenon can be logically explained by the fact that initially, Pb(II) and Cu(II) can be simultaneously adsorbed because of the high availability of active binding sites and as the sorption continues, the competition between these two metal ions occur due to the gradual occupancy of active sites. Previous studies [8,9], using the same materials conditioned under the form of spherical beads, confirmed the selectivity sequence of Pb(II) over Cu(II) onto these sorbents in batch experiments: when applying Cu-loaded beads for Pb(II) removal, a large amount of Cu(II) was released into the solution, while reciprocally, much less Pb(II) release was observed. Therefore, these carboxyl groups-based sorbents have a higher affinity for Pb(II) over Cu(II) [9,46], and when the available sorption sites are gradually occupied, Pb(II) started to replace Cu(II), leading to the release of Cu(II) into the effluent. The Cu(II) release behavior is less marked in the case of AB foams, which should be attributed to the presence of amine groups on proteins, whose higher sorption capacity (mmol g^−1^) for Cu(II) over Pb(II) has been already reported [47,48]. It is noteworthy that the molar ratio of Pb(II)/Cu(II) on the sorbents is 3.82 for alginate and 3.11 for AB foams after sorption process.

The desorption of Pb(II) and Cu(II) from bi-metallic loaded sorbents was conducted to evaluate the possibility of simultaneously desorbing the metals from loaded foams or selectively recovering the metals. The eluate curves shown in Figure 7b,d suggest that Pb(II) and Cu(II) are desorbed at the same time. Most of Cu(II) can be desorbed at the first 200 mL while after that, Pb(II) is continuously released from the foams: the Pb(II) desorbed amount is 0.12 mmol for alginate and 0.07 mmol for AB foams when using 200 mL of desorption agent, much higher than the relevant values for Cu(II) (i.e., 0.03 and 0.02 mmol, respectively). Consequently, the efficiency of Pb(II) desorption (not shown in the figure) is 82% for alginate and 84% for AB foams after the whole desorption process, much higher than the values for Cu(II) (i.e., 59% and 65%, respectively). Moreover, the desorption efficiency of Cu(II) in the Pb(II)-Cu(II) is much lower than that in mono-metal system (Figure S5b, see Supplementary Materials). This can be explained by the fact that the Cu(II) desorbed by Pb(II) (through ion-exchange) in the sorption process should be the part which is the easiest to be desorbed, leaving those bound firmly or occupying the inner sorption sites, which are rather difficult to be released. The molar ratio of Pb(II)/Cu(II) in the accumulated eluate is 5.4 and 3.9 for alginate and AB foams, respectively, which are only slightly higher than those originally loaded. The results suggest although both alginate and AB foams have a preference for Pb(II) over Cu(II), neither of them could selectively separate Pb(II) in Pb(II)-Cu(II) system through sorption and/or desorption processes.

The results in this study show that the brown algal (*L. digitata*) foams can be easily applied in a fixed-bed system with marked removal performance for Pb(II) (even from Pb(II)-Cu(II) co-existing solutions). Further research needs to be conducted to functionalize the foams (incorporating amine or sulfonic groups) to improve sorption capacity of these materials for a wide range of metals and/or to be more selective in solutions of increasing complexity that could contain anions, metal complexes, etc. Another factor worthy of exploring is to change the shape of the sorbents to widen their application (such as cylindrical foams with high percolating characteristics that can be applied in a tube-like monolith, currently under investigation). Moreover, other ions such as Ca(II), Mg(II), K(I) and Na(I) are commonly found in Pb(II)-containing industrial effluents [49,50], and some of them are very likely to compete with Pb(II) for the binding sites or degrade the biopolymer (the formation of soluble Na-alginate or K-alginate, etc.). Although the measurement of pH_PZC_, in which 0.1 M NaCl was the background electrolyte, shows that the foams are still stable when the Na(I) concentration is 0.1 M, experiments are still highly needed for better understanding the impact of these kinds of metal ions with a wider concentration range on both the stability and the sorption performance of the sorbents. The objective would be to define the limits of application of this kind of materials.

## 3. Materials and Methods

### 3.1. Materials

Marine brown alga thalli, *Laminaria digitata*, (*Heterokonta* (phylum), *Laminariales* (order), *Laminariaceae* (family)) was supplied by SETALG (Pleubian, France). The biomass was firstly washed, dried, grinded, and sieved (<250 µm). The method reported by McHugh [51], and modified by Bertagnolli et al. [52] was applied for characterizing the alginate content in the biomass: *L digitata* contains about 31% (*w*/*w*) of alginate. Alginate was obtained from FMC BioPolymer (USA) (commercial reference: Protanal 200S). The fractions of mannuronic (M) and guluronic (G) acids in the alginate were determined by NMR analysis [53]. The M/G fraction of alginate extracted from *L. digitata* was 0.62/0.38, while this of the alginate reference material (i.e., Protanal 200S) was 0.37/0.63.

Copper and lead nitrate salts were supplied by Sigma-Aldrich (Taufkirchen, Germany). Other reagents such as sodium carbonate, formic acid and calcium chloride were purchased from Chem-Lab NV (Zedelgem, Belgium).

### 3.2. Synthesis of Macroporous Foams

The foams were prepared by ionotropic gelation. The procedure (described in Supplementary Materials) has been reported in a previous study [54].

### 3.3. Characterization of Materials

For characterization, the Pb(II)-loaded foams were prepared by contacting a Pb(II) solution (pH: 4; v: 500 mL; C_0_: 0.25 mmol Pb L^−1^) with 50 mg of foams for 48 h. Pb(II)-desorbed foams were obtained using 50 mL of 2 M HCl/0.05 M CaCl_2_ as the desorption agent for 6 h. For the recycled sorbents, the sorbents were obtained from the sorption/desorption cycle experiments (conditions shown as below).

FT-IR spectrometry analysis was performed in the range 4000–400 cm^−1^ using an FTIR-ATR (Attenuated Total Reflectance tool) Bruker VERTEX70 spectrometer (Bruker, Germany).

Analysis by scanning electron microscopy (SEM) and SEM-EDX (SEM coupled with energy dispersive X-ray diffraction analysis) were performed using an environmental scanning electron microscope Quanta FEG 200 (FEI France, Thermo Fisher Scientific, Mérignac, France), equipped with an Oxford Inca 350 energy dispersive X-ray micro-analyzer (Oxford Instruments France, Saclay, France).

### 3.4. Recirculation Mode

#### 3.4.1. Sorption Isotherms

The foams were fixed in a filtration holder (Swinnex, Ø 25 mm, Millipore, Merck Chimie SAS, Fontenay sous Bois, France). The solutions containing different concentrations of Pb(II) were pumped through the reactive foams via a recirculation mode for 48 h. The dosage (D) was 0.4 g L^−1^ (dry weight) and feed Pb(II) concentration (i.e., initial metal concentration, C_0_, mmol Pb L^−1^) varied between 0.05 and 1.5 mmol Pb L^−1^. The residual metal concentration (C_eq_, mmol Pb L^−1^), after filtration, was analyzed using inductively coupled plasma atomic emission spectrometry (ICP-AES, JY Activa M, Jobin-Yvon, Horiba, Longjumeau, France). The sorption capacity (q, mmol Pb g^−1^) was calculated by the mass balance equation: q_eq_ = (C_0_ − C_eq_)/D.

#### 3.4.2. Sorption Kinetics

The effect of flow rate (5, 25 and 45 mL min^−1^, i.e., superficial velocities: 0.6, 3 and 5.5 m h^−1^) on uptake kinetics was studied. The sorbent dosage for uptake kinetics was set at 0.2 g L^−1^ for alginate and 0.4 g L^−1^ for AB foams (Pb(II) concentration was 0.5 mmol Pb L^−1^).

#### 3.4.3. Desorption Kinetics

The Pb-loaded foams were obtained from sorption kinetics (flow rate: 25 mL min^−1^). They were contacted with the desorption agent (2 M HCl/0.05 M CaCl_2_ solution) in the recirculation mode (flow rate: 25 mL min^−1^). Samples were collected at different contact times for plotting desorption kinetics.

#### 3.4.4. Sorbent Reuse

For the study of metal desorption and sorbent reuse, a washing step (pumping 50 mL of demineralized water) was intercalated between successive desorption and sorption phases; desorption efficiency was calculated dividing the amount of metal released during the desorption step by that adsorbed on the sorbent at the preceding step.

Based on the results obtained from batch experiments using alginate and algal beads [18], the pH value of 4.0 was used for all the experiments in this study and all the experiments in this study were performed at room temperature (the same for single-pass mode).

Full experimental conditions are systematically reported in the caption of the Figures.

### 3.5. Single-Pass Mode

Continuous sorption experiments were performed by pumping the solution through the foams in a one-pass mode. A sample collector was connected to the outlet to collect the samples. The saturation time (or volume) (C_effluent_/C_feed_ = 0.95) and breakthrough time (or volume) (C_effluent_/C_feed_ = 0.05) were determined.

For the study of effect of flow rate, the velocity was kept at 0.5 mL min^−1^ or 5 mL min^−1^. The feed solutions containing different concentrations (0.25, 0.5 and 1 mmol Pb L^−1^) were fed into the holder containing one foam disc (100 mg). For investigating the impact of bed depth, the flow rate was fixed at 5 mL min^−1^. The feed solutions containing different concentrations (0.25 and 0.5 mmol Pb L^−1^) were fed into the holder with one or two foam discs (i.e., column depth: 0.32 cm and 0.64 cm for alginate foams, and 0.26 cm and 0.52 cm for AB foams). For the study of desorption, the eluent used was the same as for recirculation mode (i.e., 2 M HCl/0.05 M CaCl_2_ solution).

The binary sorption of Pb(II) and Cu(II) was carried out with equimolar concentration of Pb(II) and Cu(II) (i.e., 0.5 mmol L^−1^). The flow rate was kept at 2 mL min^−1^ and the filtration bed consisted of single foam disc.

It is noted that selected sorption tests were duplicated and the standard deviation of sorption capacity was less than 0.02 mmol g^−1^ for alginate foams (q_max_ = 2.12 mmol g^−1^) and 0.01 mmol g^−1^ for AB foams (q_max_ = 1.12 mmol g^−1^). During the experiments, the alginate foams were once used up, the alginate foams prepared at different times were compared in one-pass mode at 0.5 mL min^−1^ and the breakthrough curves are presented at the end of the Supplementary Materials section. The plots almost overlapped; this clearly demonstrates that the sorption properties and the manufacturing of the sorbents are reproducible.

### 3.6. Modeling of Sorption Isotherms and Uptake Kinetics

Pseudo-first order rate equation (PFORE) and pseudo-second order rate equation (PSORE) were used for modeling sorption kinetics, and sorption isotherms were fitted by the Langmuir equations (linear or non-linear regression analysis). Breakthrough curves were fitted by the Thomas and Yan models. All the equations are summarized in the Supplementary Materials section.

## 4. Conclusions

Alginate and algal biomass (AB) macroporous foams were immobilized in a filtration holder and tested in fixed-bed column system for Pb(II) sorption using recirculation or one-pass modes. The recirculation mode confirms that sorption isotherms can be well fitted by linear Langmuir model and the pseudo-second order rate equation successfully predicts both sorption and desorption kinetics data for both alginate and AB foams. In addition, increasing the flow rate from 5 to 45 mL min^−1^ significantly improved the sorption kinetics of Pb(II) onto alginate, while for AB foams, the effect was less marked, especially when the velocity was more than 25 mL min^−1^. The results obtained in sorption/desorption cycles demonstrated that the reuse of the sorbents is feasible (at least for 9 cycles). One-pass mode shows that for minimizing the effects of resistance to intra-particle diffusion it is necessary using a slow flow rate. In the case of AB foams the impact of flow rate is less marked. It is proposed that the presence of cellulose-like fibers in the biomass improves the diffusion performance, which, in turn, decreases the effect of flow rate. In addition, the effect of Cu(II) on Pb(II) recovery from binary solutions was also studied in terms of both sorption and desorption. The results confirm the trends previously observed with similar materials conditioned as beads: both metal ions can be sorbed (and desorbed) simultaneously with a marked preference for Pb(II) over Cu(II); though this preference is not sufficient for selectively separating the two metal ions. These results show the potential of this new conditioning of algal biomass (without addition of encapsulating biopolymer) for applications in metal recovery (fixed-bed system) or for providing a securing dispositive for confining metal ions in case of accidental dumping of contaminated effluent, as a highly reactive and enhanced-percolating filtrating media.

## Figures and Tables

**Table 1:** Uptake kinetics—Modeling parameters for PFORE and PSORE (flow rate = 45 mL min^−1^).

Model	Parameter	Alginate	AB
Experiment	q_eq,exp_ (mmol Pb g^−1^)	1.88	1.03
PFORE	q_eq,calc_ (mmol Pb g^−1^)	1.76	0.93
	k_1_ × 10^3^ (min^−1^)	28.25	34.09
	R^2^	0.979	0.972
PSORE	q_eq,calc_ (mmol Pb g^−1^)	1.89	1.00
	k_2_ × 10^3^ (g mmol^−1^ min^−1^)	20.09	44.21
	R^2^	0.996	0.989

**Table 2:** Parameters of breakthrough curves obtained from different input metal concentration at different flow rates.

Velocity (mL min^−1^)	C_0_ (mmol Pb g^−1^)	t_b_ (min)		t_s_ (min)		V_b_ (mL)		V_s_ (mL)	
		Alginate	AB	Alginate	AB	Alginate	AB	Alginate	AB
0.5	0.25	880	160	2480	2400	440	80	1240	1200
	0.5	400	80	1760	1440	200	40	880	720
	1	120	<40	920	760	60	<20	460	380
5	0.25	48	8	320	280	240	40	1600	1400
	0.5	24	8	216	144	120	40	1080	720
	1	<4	<4	100	88	<20	<20	500	440

Note: C_0_, feed Pb(II) concentration; t_b_, breakthrough time; t_s_, saturation time; V_b_, breakthrough volume; V_s_, saturation volume.

## Supplementary Materials

The following are available online at www.mdpi.com/1660-3397/15/10/315/s1, description of synthesis procedure for macroporous foams, reminder on (a) modeling of sorption isotherms and uptake/desorption kinetics in batch experiments, (b) modeling of breakthrough curves. Figure S1: EDX analysis of raw, Pb(II)-loaded, Pb(II)-desorbed and recycled alginate and AB foams, Figure S2: FT-IR spectra of raw, Pb(II)-loaded, Pb(II)-desorbed and recycles alginate (a) and AB foams (b), Figure S3: Sorption isotherms of Pb(II) onto Alginate and AB foams (Dose: 0.4 g L^−1^, initial metal concentration varied between 0.05 and 1.5 mmol Pb L^−1^, solution pH: 4, contact time: 48 h, room temperature), Figure S4: Comparison of the experimental and predicted breakthrough curves obtained at various conditions according to the Adams–Bohart model, Figure S5: (a) Breakthrough curves and (b) eluate curves of Cu(II) on/from alginate and AB foams (C_0_: 0.5 mmol Cu L^−1^; flow rate: 2 mL min^−1^; solution pH: 4; mass: 100 mg), Figure S6: Breakthrough curves for Pb(II) sorption onto alginate foams prepared at different times (pH: 4; sorbent mass: 100 mg), Table S1: Determination of the pH_ZPC_, porosity and bulk density of the sorbents, Table S2: Experimental frequencies of the bands observed for raw, Pb-loaded, Pb-desorbed and recycled alginate and AB foams. Unit: cm^−1^, Table S3: Sorption isotherms—Modeling parameters for Langmuir (non-linear and linear), Table S4: Desorption kinetics—Modeling parameters for PFORE and PSORE, Table S5: The Thomas and Yan models constants for Pb(II) onto alginate and AB foams at flow rates of 0.5 mL min^−1^ and 5 mL min^−1^ (C_0_: 0.5 mmol Pb L^−1^, number of foam: 1, pH: 4, room temperature), Table S6: Parameters of the Adams–Bohart model for Pb(II) sorption onto alginate and AB foams at different feed concentration and bed height (Flow rate: 5 mL min^−1^, pH: 4, room temperature).
