# Supplementary material for: Algal Foams Applied in Fixed-Bed Process for Lead(II) Removal Using Recirculation or One-Pass Modes
Source: Mar Drugs. 2017 Oct 17;15(10):315. doi: 10.3390/md15100315 (PMC5666423; doi:10.3390/md15100315)
Supplement: Supplementary file 1 [file marinedrugs-15-00315-s001.pdf]

marine drugs

Supplementary Materials

Algal foams applied in fixed-bed process for lead(II) removal using recirculation or one-pass modes

Shengye WANG[1,*] Thierry VINCENT[1], Catherine FAUR[2] and Eric GUIBAL[1,*]

1 Ecole des mines d'Alès, Centre des Matériaux des Mines d'Alès, Pôle Matériaux Polymères Avancés, 6 avenue de Clavières, F-30319 Alès cedex, France ; Thierry.Vincent@mines-ales.fr
2 Institut Européen des membranes, IEM (UMR-5635, Université de Montpellier, ENSCM, CNRS), Place Eugène Bataillon, 34095 Montpellier cedex 5, France; Catherine.Faur@umontpellier.fr
* Correspondence: Shengye.Wang@mines-ales.fr; Tel.: +33-076-142-9171, Eric.Guibal@mines-ales.fr; Tel.: +33-046-678-2734

1. Synthesis of macroporous foams

Ionotropic gelation was applied for the preparation of pure alginate discs but also for the gelation of alginate extracted from algal biomass (for AB foams). A given volume (i.e., 600 mL) of alginate solution (1%, w/w) or suspension (AB) was first homogeneously mixed with 20 mL of $CaCO_3$ suspension (1%, w/w). Then the mixture was dipped into the molds (diameter: 50 mm, height: 4.0 mm), stored in a freezer at −80 °C for 1 h and freeze-dried (−52 °C, 0.1 mbar, 48 h). The dried discs were immersed in a solution containing both $CaCl_2$ (1%, w/w) and formic acid (1%, v/w) under shaking (20 rpm) for 24 h, washed 4 times with 4 L (in total) of pure water and freeze-dried (−52 °C, 0.1 mbar, 24 h).

Specifically, the algal biomass suspension was prepared by adding 15 g of *L. digitata* (dry) and 3 g of Na_2CO_3 into 576 mL of pure water. The mixture was then maintained at 50 °C for 24 h. All other steps were similar to the procedure followed for alginate material.

2. Modeling of sorption isotherms and uptake/desorption kinetics in batch experiments

Uptake kinetics have been modeled using both the pseudo-first order rate equation (PFORE) [1], and the pseudo-second order rate equation (PSORE) [2].

PFORE: $$q(t) = q_{eq}(1 - e^{-k_1 t}) \quad (1)$$

PSORE: $$q(t) = \frac{q_{eq}^2 k_2 t}{1 + q_{eq} k_2 t} \quad (2)$$

with; $q_{(t)}$ and q_{eq} (mmol g^{-1}): sorption capacities adsorbed at t and at equilibrium, respectively.

The parameters k_1 and k_2 are the apparent rate coefficients of PFORE (min^{-1}) and PSORE (g $mmol^{-1}$ min^{-1}), respectively.

It is noteworthy that the PFORE and PSORE models have been initially designed for the description of reaction kinetics in homogeneous systems. These equations are commonly used now for fitting uptake kinetics in heterogeneous systems. This means that the kinetic parameters (k_1 and k_2) that can be derived from the mathematical fits should be considered as apparent rate coefficients that take into account the specific limitations associated to the mechanisms of resistance to diffusion (film diffusion, intraparticle diffusion).

Desorption kinetics were also fitted by PSORE:

$$q_t = q_0 - \frac{(q_0 - q_e)^2 k_{2d} t}{1 + (q_0 - q_e) k_{2d} t} \quad (3)$$

Where k_{2d} is rate constant of pseudo-second-order desorption (g $mmol^{-1}$ min^{-1}), q_0 is the initial sorption capacity before desorption process (mmol g^{-1}) and q_e is the equilibrium sorption capacity after desorption process (mmol g^{-1}). The parameters of PFORE and PSORE equations (i.e., q_{eq}, k_1, k_2 and k_{2d}) were determined by non-linear regression analysis using Mathematica software.

Sorption isotherms describe the distribution of metal ions between the liquid and the solid phases (when varying metal concentration in the system). They plot sorption capacity (i.e., q_{eq}) *vs.* residual metal concentration (i.e., C_{eq}). Non-linear (4) and linear (5) Langmuir equations was used to fit the experimental data.

Non-linear Langmuir equation: $$q_{eq} = \frac{q_m b C_{eq}}{1 + b C_{eq}} \quad (4)$$

Linear Langmuir equation: $$\frac{C_e}{q_{eq}} = \frac{C_e}{q_m} + \frac{1}{q_m \times b} \quad (5)$$

The parameters of Non-linear Langmuir equation were determined by non-linear regression analysis using Mathematica software.

2. Modeling of breakthrough curves

The data obtained in column in one-pass mode were modeled by the Thomas equation, which is one of the most general and widely used methods in column performance theory. The expression by Thomas [3] for a sorption column is given as follows:

$$\frac{C_t}{C_0} = \frac{1}{1 + \exp(K_{Th} q_{Th} m / F - C_0 t)} \quad (6)$$

where K_{Th} is the Thomas rate constant (L min^{-1} $mmol^{-1}$), q_{Th} is equilibrium sorption capacity for Pb(II) (mmol g^{-1}), m is the mass of sorbent in the column (g), C_0 is the feed Pb(II) concentration (mmol Pb L^{-1}), C_t the effluent concentration at time t (mM) and F is the flow rate (L min^{-1}).

Yan model [4] helps to overcome some of the drawbacks associated to Thomas model, like serious deficiency in predicting the effluent concentration during the second phase phase of the sorption process (i.e., close to the saturation of the breakthrough curve). The equation is as follows:

$$\frac{C_t}{C_0} = 1 - \frac{1}{1 + (\frac{F C_0 t}{q_Y m})^{a_Y}} \quad (7)$$

where, a_Y is the Yan's model constant (dimensionless), q_Y is the maximum sorption capacity (mmol g^{-1}), m is the mass of adsorbent (g), F is flow rate (L min^{-1}) and t is the time (min), C_t and C_0 are the concentrations of effluent and feed (mmol L^{-1}), respectively.

The Adams–Bohart model was used for the description of the initial part of the breakthrough curve:

$$\frac{C_t}{C_0} = \exp(k_{AB}C_0t - k_{AB}N_0\frac{Z}{v}) \quad (8)$$

where k_{AB} is the kinetic constant (L mmol^{-1} min^{-1}), v is the linear velocity calculated by dividing the flow rate by the column section area (cm min^{-1}), Z is the bed depth of column and N_0 is the saturation concentration (mmol L^{-1}).

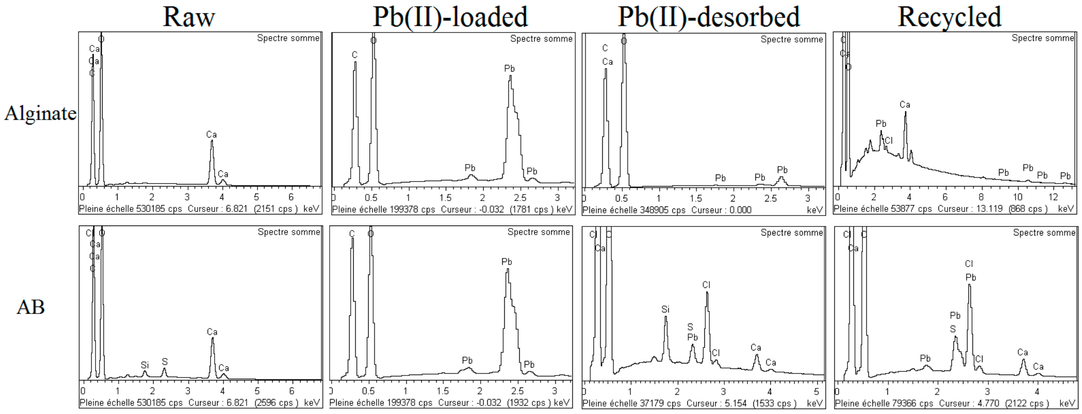

Figure S1. EDX analysis of raw, Pb(II)-loaded, Pb(II)-desorbed and recycled alginate and AB foams.

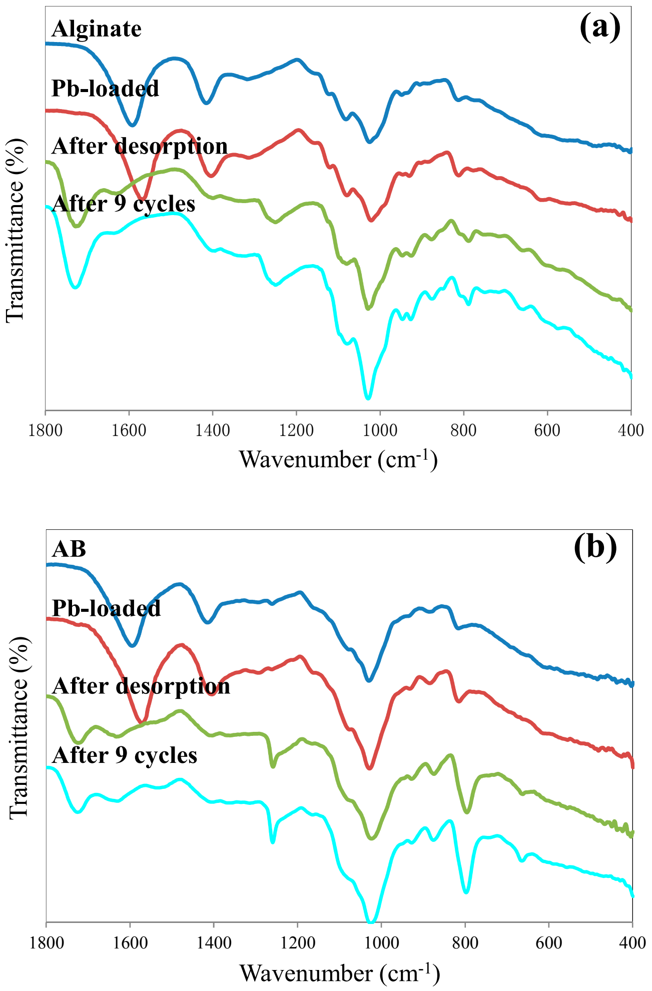

Figure S2. FT-IR spectra of raw, Pb(II)-loaded, Pb(II)-desorbed and recycles alginate (a) and AB foams (b).

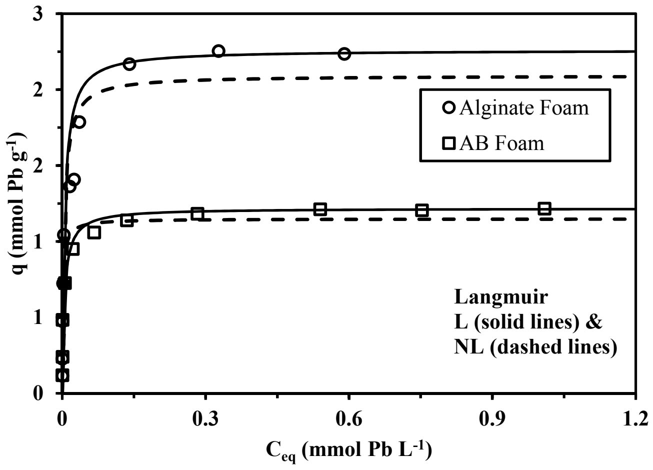

Figure S3. Sorption isotherms of Pb(II) onto alginate and AB foams (Dose: 0.4 g L^{-1}, initial metal concentration varied between 0.05 and 1.5 mmol Pb L^{-1}, solution pH: 4, contact time: 48 h, room temperature).

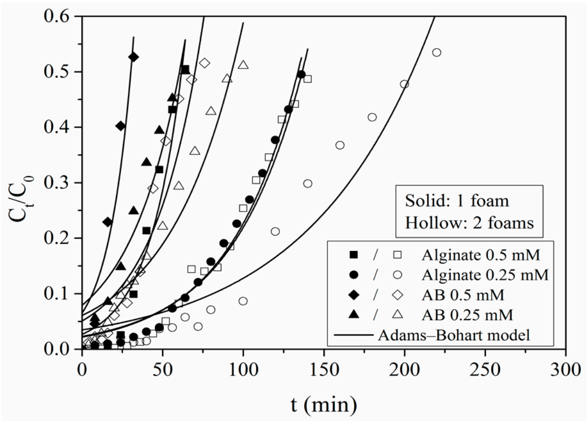

Figure S4. Comparison of the experimental and predicted breakthrough curves obtained at various conditions according to the Adams–Bohart model.

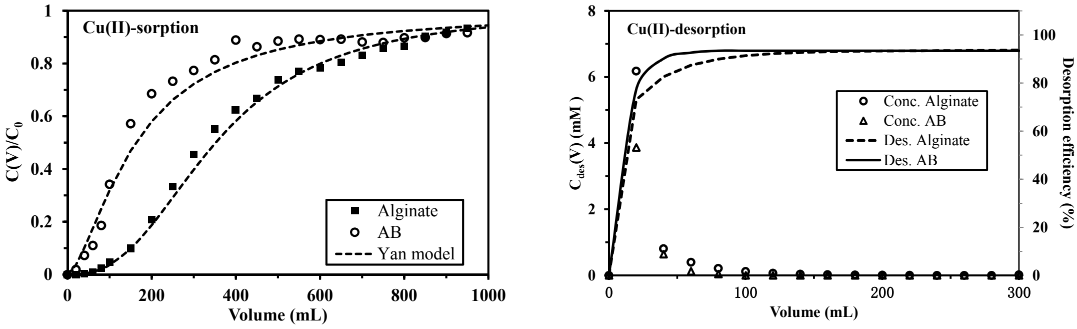

a b

Figure S5. (a) Breakthrough curves and (b) eluate curves of Cu(II) on/from alginate and AB foams (C_0: 0.5 mmol Cu L^{-1}; flow rate: 2 mL min^{-1}; solution pH: 4; mass: 100 mg).

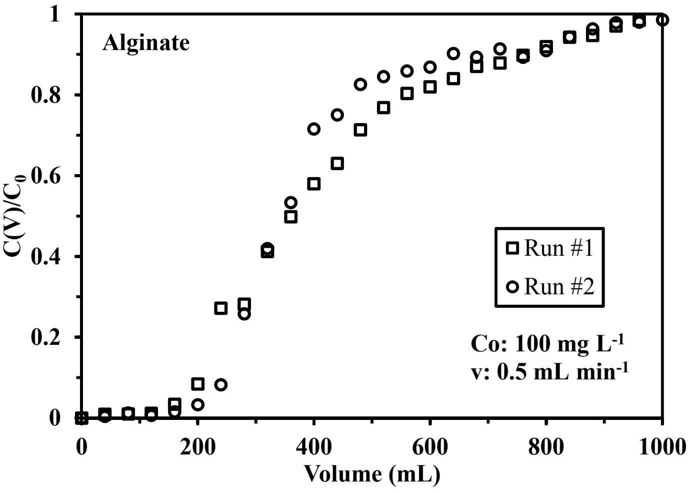

Figure S6. Breakthrough curves for Pb(II) sorption onto alginate foams prepared at different times (pH: 4; sorbent mass: 100 mg).

5. Tables

Table S1: Determination of the pH_{PZC}, porosity and bulk density of the sorbents.

Sorbents	Alginate	AB
Porosity (%)	90.9±0.4	93.2±1.0
Bulk density (g/cm^3)	0.031±0.0002	0.032±0.0004
pH_{PZC}[a]	4.45	5.09
Thickness (cm)	0.319±0.003	0.257±0.001

[a]: The pH_{PZC} (point of zero charg) of the sorbents were determined by the pH drift method – pH variation after contact of the sorbents with 0.1 M NaCl solutions and variable initial pH [5]. The pH_{PZC} corresponds to the pH value where pH_{eq}=pH_0, and to the pH conditions for charge neutralization at the surface of the materials.

marine drugs

MDPI

Table S2. Experimental frequencies of the bands observed for raw, Pb-loaded, Pb-desorbed and recycled alginate and AB foams. Unit: cm^{-1}.

Vibration	In reference	Alginate				AB				Reference
		Raw	Pb-loaded	Pb-desorbed	Recycled	Raw	Pb-loaded	Pb-desorbed	Recycled	
–OH stretching	3500-3000	3240	3209	3386	3404	3260	3276	3285	3335	[6]
CH stretching	2928	2928	2927	2925	2923	2920	2923	2961	2961	[7]
Carboxylic acid C=O stretching	1730	–	–	1728	1728	–	–	1723	1725	[8]
COO– asymmetric stretching	1650-1580	1592	1568	–	–	1595	1573	1633	1630	[9]
COO– symmetric stretching	1429	1415	1404	1399	1398	1415	1404	–	–	[10]
CH_3 symmetric bending	1260	–	–	1253	1250	–	–	1259	1260	[11]
C–O–C antisym. stretching	1025	1025	1021	1028	1029	1030	1028	1023	1025	[12]
CH_3 rocking	810	813	812	788	789	816	814	796	797	[13]

Table S3: Sorption isotherms – Modeling parameters for Langmuir equation (non-linear and linear).

Model	Parameter	Alginate	AB
Experiment	$q_{eq,exp}$ (mmol Pb g^{-1})	2.24	1.22
Langmuir (Non-linear)	$q_{eq,calc}$ (mmol Pb g^{-1})	2.09	1.15
	b (L mmol^{-1})	232.3	582.7
	R^2	0.900	0.949
Langmuir (Linear)	$q_{eq,calc}$ (mmol Pb g^{-1})	2.26	1.22
	b (L mmol^{-1})	193.0	204.9
	R^2	0.999	0.999

Table S4. Desorption kinetics – Modeling parameters for PFORE and PSORE.

Sorbent	$k_{2d}\times 10^2$ (g mmol^{-1} min^{-1})	q_0 (mmol g^{-1})	q_e (mmol g^{-1})	R^2
Alginate	2.86	1.89	0.21	0.992
AB	22.9	1.01	0.11	0.944

Note: q_0– amount loaded per gram sorbent before desorption; q_e–metal amount loaded per gram sorbent after desorption.

Table S5. The constants of Thomas and Yan models for Pb(II) onto alginate and AB foams at flow rates of 0.5 mL min^{-1} and 5 mL min^{-1} (C_0: 0.5 mmol Pb L^{-1}, number of foam: 1, pH: 4, room temperature).

Velocity (mL min^{-1})	Sorbent	Experiment	Thomas model			Yan model		
		$q_{e,exp}$ (mmol g^{-1})	$k_{Th}\times 10^3$ (L min^{-1} mmol^{-1})	q_{Th} (mmol g^{-1})	R^2	a_Y	q_Y (mmol g^{-1})	R^2
5	Alginate	2.11	67.9	2.49	0.73	2.99	1.86	0.95
	AB	1.08	72.1	0.95	0.87	2.07	0.80	0.97
0.5	Alginate	2.12	8.94	2.47	0.88	3.85	1.99	0.95
	AB	1.12	8.47	1.13	0.82	2.15	0.85	0.98

Table S6. Parameters of the Adams–Bohart model for Pb(II) sorption onto alginate and AB foams at different feed concentration and bed height (Flow rate: 5 mL min^{-1}, pH: 4, room temperature).

Sorbent	C_0 (mmol Pb L^{-1})	Bed height (cm)	k_{AB}×10^3 (L min^{-1} mmol^{-1})	N_0 (mmol L^{-1})	R^2
Alginate	0.5	0.32	94.2	122.5	0.93
		0.64	44.2	136.2	0.96
	0.25	0.32	96.1	126.7	0.98
		0.64	51.4	104.1	0.92
AB	0.5	0.26	143.1	74.9	0.89
		0.52	66.1	88.4	0.89
	0.25	0.26	129.5	76.9	0.90
		0.52	88.1	62.4	0.93

References

1. Lagergren, S. About the theory of so-called adsorption of soluble substances. *Kungliga Svenska Vetenskapsakademiens* **1898**, *24*, 1-39.
2. Ho, Y.S.; McKay, G. Pseudo-second order model for sorption processes. *Process Biochemistry* **1999**, *34*, 451-465.
3. Thomas, H.C. Heterogeneous ion exchange in a flowing system. *J. Am. Chem. Soc.* **1944**, *66*, 1664-1666.
4. Yan, G.; Viraraghavan, T.; Chen, M. A new model for heavy metal removal in a biosorption column. *Adsorpt. Sci. Technol.* **2001**, *19*, 25-43.
5. Wang, S.; Vincent, T.; Roux, J.-C.; Faur, C.; Guibal, E. Innovative conditioning of algal-based sorbents: Macro-porous discs for palladium sorption. *Chem. Eng. J.* **2017**, *325*, 521-532.
6. Schiewer, S.; Balaria, A. Biosorption of Pb^{2+} by original and protonated citrus peels: Equilibrium, kinetics, and mechanism. *Chem. Eng. J.* **2009**, *146*, 211-219.
7. Naebe, M.; Wang, J.; Amini, A.; Khayyam, H.; Hameed, N.; Li, L.H.; Chen, Y.; Fox, B. Mechanical property and structure of covalent functionalised graphene/epoxy nanocomposites. *Sci. Rep.* **2014**, *4*, 4375.
8. Akhtar, M.; Iqbal, S.; Kausar, A.; Bhanger, M.; Shaheen, M.A. An economically viable method for the removal of selected divalent metal ions from aqueous solutions using activated rice husk. *Colloids Surf., B* **2010**, *75*, 149-155.
9. Luo, J.; Wang, L.; Mott, D.; Njoki, P.N.; Kariuki, N.; Zhong, C.-J.; He, T. Ternary alloy nanoparticles with controllable sizes and composition and electrocatalytic activity. *J. Mater. Chem.* **2006**, *16*, 1665-1673.
10. Theras, J.E.M.; Kalaivani, D.; Jayaraman, D.; Joseph, V. Growth and spectroscopic, thermodynamic and nonlinear optical studies of l-threonine phthalate crystal. *J. Cryst. Growth* **2015**, *427*, 29-35.
11. Wang, L.; Ji, Q.; Glass, T.; Ward, T.; McGrath, J.; Muggli, M.; Burns, G.; Sorathia, U. Synthesis and characterization of organosiloxane modified segmented polyether polyurethanes. *Polymer* **2000**, *41*, 5083-5093.

12. Lawrie, G.; Keen, I.; Drew, B.; Chandler-Temple, A.; Rintoul, L.; Fredericks, P.; Grøndahl, L. Interactions between alginate and chitosan biopolymers characterized using ftir and xps. *Biomacromolecules* **2007**, *8*, 2533-2541.
13. Morent, R.; De Geyter, N.; Leys, C.; Gengembre, L.; Payen, E. Comparison between XPS-and FTIR-analysis of plasma-treated polypropylene film surfaces. *Surf. Interface Anal.* **2008**, *40*, 597-600.

© 2017 by the authors. Submitted for possible open access publication under the terms and conditions of the Creative Commons Attribution (CC BY) license (http://creativecommons.org/licenses/by/4.0/).
